# Authentication of African green monkey cell lines using human short tandem repeat markers

**DOI:** 10.1186/1472-6750-11-102

**Published:** 2011-11-07

**Authors:** Jamie L Almeida, Carolyn R Hill, Kenneth D Cole

**Affiliations:** 1 National Institute of Standards and Technology, Biochemical Science Division, Bioassay Methods Group, 100 Bureau Drive MS8312, Gaithersburg, MD 20899, USA; 2 National Institute of Standards and Technology, Biochemical Science Division, Applied Genetics Group, 100 Bureau Drive MS8316, Gaithersburg, MD 20899, USA

## Abstract

**Background:**

Tools for authenticating cell lines are critical for quality control in cell-based biological experiments. Currently there are methods to authenticate human cell lines using short tandem repeat (STR) markers based on the technology and procedures successfully used in the forensic community for human identification, but there are no STR based methods for authenticating nonhuman cell lines to date. There is significant homology between the human and vervet monkey genome and we utilized these similarities to design the first multiplex assay based on human STR markers for vervet cell line identification.

**Results:**

The following STR markers were incorporated into the vervet multiplex PCR assay: D17S1304, D5S1467, D19S245, D1S518, D8S1106, D4S2408, D6S1017, and DYS389. The eight markers were successful in uniquely identifying sixty-two vervet monkey DNA samples and confirmed that Vero76 cells and COS-7 cells were derived from Vero and CV-1 cells, respectively. The multiplex assay shows specificity for vervet DNA within the determined allele range for vervet monkeys; however, the primers will also amplify human DNA for each marker resulting in amplicons outside the vervet allele range in several of the loci. The STR markers showed genetic stability in over sixty-nine passages of Vero cells, suggesting low mutation rates in the targeted STR sequences in the Vero cell line.

**Conclusions:**

A functional vervet multiplex assay consisting of eight human STR markers with heterozygosity values ranging from 0.53-0.79 was successful in uniquely identifying sixty-two vervet monkey samples. The probability of a random match using these eight markers between any two vervet samples is approximately 1 in 1.9 million. While authenticating a vervet cell line, the multiplex assay may also be a useful indicator for human cell line contamination since the assay is based on human STR markers.

## Background

Misidentification of cell lines has been a continuing problem in academia and industry for decades [1-6]. The most common causes of cell misidentification include mislabeling, contamination of cell lines (i.e. feeder cells used in cocultivation of human stem cells), and xenografting (transplanting living cells, tissues, or organs from one species to another) [7,8]. Erroneous data have been published based on results derived from misidentified cells [9] and only recently has an active push for cell authentication been proposed. Nardone *et al*. attempted to raise awareness of this issue in an open letter to Secretary Michael O. Leavitt at the U.S. Department of Health and Human Services [10-12]. Subsequently, NIH has urged researchers to authenticate cell lines in the "Notice Regarding Authentication of Cultured Cell Lines", an addition to their Guidelines for Research [13]. As a result, more scientific journals are now requiring proof of cell line authentication for submission of manuscripts [14]. Major cell repositories are confirming cell line identity by incorporating these data into a large comprehensive international reference database [15]. There are also extensive lists of documented misidentified cells available to educate cell culture users [3,16]. In 2009, a working group was formed to develop a standard for authentication of human cells along with a database of short tandem repeat (STR) profiles for human cell lines [14].

Recently, the use of short tandem repeat markers has been recommended for cell line authentication and these methods are currently being used to identify human cell lines [17,18]. The most successful STR markers used in human identification have tetranucleotide repeats for reduced stutter product formation and offer a narrow allele size range that is beneficial for multiplexing [19]. Di- and trinucleotide repeats tend to have higher stutter due to strand slippage during PCR and these markers are generally avoided [20]. Many commercially available kits for human STR profiling target these tetranucleotide repeat regions, give rapid results, and have been well characterized in the forensic community http://www.cstl.nist.gov/biotech/strbase[21]. Additional human STR markers have also been developed for use in complex multiplex assays to aid in human identification and could also be useful in human cell line authentication [22].

Cell authentication targeting STR loci can also be expanded to nonhuman cell lines based on genome availability. This study describes a method to identify cell lines derived from the vervet monkey (*C. aethiops sabaeus*), commonly known as the African green monkey. Vero cells isolated from the kidney of an African green monkey in 1962 are commonly used for virus amplification, vaccine development, and cytotoxicity assays [23-29]. It has been shown that humans and vervets share homology in their STR regions enabling the use of human STR markers for vervet identification [30,31]. The genome for the vervet monkey has not been fully sequenced; however the completed vervet reference sequence is available through The Vervet Functional Genome Project (VFGP). The VFGP is a collaborative effort between researchers at UCLA and McGill University and the Genome Québec Innovation Centre. Data has been published from the collaborative group describing concordance between vervet and known human STR markers [31]. Utilizing known human STR markers and concordant regions found in the vervet reference sequence, we have developed a multiplex assay containing eight human STR markers that successfully genotyped sixty-two vervet genomic DNA samples from monkeys and commonly used vervet cell lines.

## Methods

### Selection of loci

STR markers with high heterozygosity values are beneficial in a multiplex assay as they increase the variability and reduce the chance of a random sample match [19]. This was an important parameter because fifty-nine human STR markers were screened against a panel of vervet monkey DNA samples. Eight STR markers were selected based on the following criteria: 1) heterozygosity values greater than 50%, 2) locus must have at least 4 unique alleles, 3) locus must be present in every sample tested, 4) locus must contain a tetranucleotide repeat, and 5) primers for each marker must amplify products in a functional multiplex. Eight human loci (D8S1106, D1S518, D6S1017, D17S1304, D4S2408, D5S1467, D19S245, and DYS389) met the criteria listed above and were incorporated into the vervet multiplex. D17S1304, D5S1467, and D19S245 were identified in the vervet genetic linkage map published by Jasinska *et al*. [31]. D1S518, D8S1106, and DYS389 were described by Newman *et al*. in a study utilizing human STR markers for vervet parentage determination [30]. D4S2408 and D6S1017 loci are included in a 26plex assay used for identification of human DNA samples [32]. Chromosomal locations for these markers were found by searching the National Center for Biotechnology Information (NCBI) UniSTS browser [33] for human STR markers. These human chromosomal locations were used to find regions of sequence similarity within the vervet reference sequence supplied by The Vervet Functional Genome Project using the UCSC Genome Browser [34].

### Primer design

Primer3 software http://fokker.wi.mit.edu/primer3/input.htm[35] was used to design PCR primers by inputing the downloaded concordant vervet sequences from the UCSC Genome Browser. The parameters for Primer3 were set to target primers with melting temperatures of 60°C. AutoDimer software was used to assess primer-dimer interactions and hairpin structures within the primers chosen for the vervet multiplex [36]. Forward primers were labeled with one of the following fluorescent dyes at the 5' end: 6FAM™ (blue), VIC™ (green), NED™ (yellow), or PET™ (red) (Applied Biosystems, Foster, CA). In some cases, an additional guanine base (G) or a "PIGtail" sequence (GTTTCTT) was added to the 5' end of the unlabeled reverse primers to promote complete adenylation (Eurofins MWG Operon, Huntsville, AL) [37] (Table 1).

**Table 1:** Primers used for STR amplification

STR Marker	Forward Primer (5'-3')	Reverse Primer (5'-3')	Primer μM
D8S1106	[FAM]-GTTTACCCCTGCATCACTGG	GTCAGAATTGCTCATAGTGCAAGA	0.045
DYS389	[FAM]-CCAACTCTCATCTGTATTATCTATG	GTCTTATCTCCACCCACCAGA	0.200
D1S518	[VIC]-GCAGATCTTGGGACTTCTCG	GTGTGGGCAACTGCATTAGAG	0.420
D6S1017	[VIC]-CTGGCACAGGATAGGTGCTC	GATTGAACCAGATGGGAACGA	0.300
D17S1304	[NED]-ACCATGTCCTCTGGTTCTGG	GTTTCTTACAGGTGGGACTTGGTGAAA	0.040
D4S2408	[NED]-TCATTTCCATAGGGTAAGTGAAAA	GCCATGGGGATAAAATCAGA	0.200
D5S1467	[PET]-GCCTAAGGTGGTGAATTGGA	GTGCATTATTGGAGGCTTTCTC	0.060
D19S245	[PET]-GACCTGCAATCAGCCATTTT	GTTCTTGCAGTCTGTGGCTTG	0.360

Reverse primers that did not end in a guanine base at the 5'end were modified by adding an additional guanine (G) base or a "PIGtail" sequence (GTTTCTT) to the 5' end of the reverse primer (underlined) to promote complete adenylation [37]. Primer concentrations listed are final concentrations of forward and reverse primers in a 20 μL reaction volume. Primer concentrations were determined empirically based on peak height, DNA concentration, and number of cycles in the PCR program.

### DNA and cell lines

Sixty vervet monkey genomic DNA samples were obtained from The Nonhuman Primate Research Center Consortium Genome Banks at the University of Washington. These samples originated from the Vervet Research Colony which is part of the Wake Forest University Primate Center and consists of descendants from fifty-seven original founders that were imported from Saint Kitts and Nevis in the mid-1970's. It has been a closed colony since the mid-1980's. Genomic DNA from hamster, rat, gerbil, mouse, pig, baboon, rhesus, and cynomolgus monkey were obtained from Zyagen (San Diego, CA). A total of ten human genomic DNA samples from NIST SRM 2391b (components 1, 3, 4, 5, 7, 8, 9, 10), AmpFℓSTR^® ^Control DNA 007 (Applied Biosystems), and a highly characterized human DNA sample of male origin from NIST (N4124) were used for human reference samples. The following cell lines were obtained from The American Type Culture Collection (ATCC, Manassas, VA): Vero (CCL-81), Vero76 (CRL1587), COS-7 (CRL-1651), CV-1 (CCL-70), and HeLa (CCL-2) cells. Vero cells were grown in Minimum Essential Medium (MEM) Alpha (Invitrogen, Carlsbad, CA) with 10% fetal bovine serum (FBS) (Invitrogen, US origin, qualified), COS-7 and Vero76 cells were grown in Dulbecco's MEM (ATCC) with 10% FBS, and CV-1 and HeLa cells were grown in Eagle's MEM (ATCC) with 10% FBS. All cells were grown in a humidified 5% CO_2 _balanced-air atmosphere at 37°C. Vero 76, COS-7 and CV-1 cells were harvested at passage number 2 using 0.25% trypsin/0.53 mM EDTA solution (ATCC). Vero cells were harvested over a period of 6 months at passages 7, 14, 22, 35, 43, 58, 63, and 69 to study changes in the STR profile over high passage number. Trypsin activity was quenched by the addition of an equivalent volume of growth medium and one million cells from each cell line were counted using the Multisizer 3 Coulter Counter (Beckman Coulter, Brea, CA). The Wizard DNA Extraction kit (Promega, Madison, WI) was used to isolate DNA from harvested cells. DNA was quantified using a Nanodrop 1000 (Thermo Scientific, Wilmington, DE) at an absorbance of 260 nm.

### PCR amplification

PCR amplification was performed on a Gene Amp 9700 or Veriti thermal cycler (Applied Biosystems, Foster City, CA). The reaction mixture of 20 μL contained 6 ng of vervet DNA, 1× GeneAmp PCR Gold buffer (Applied Biosystems), 2 μM MgCl_2 _(Applied Biosystems), 250 μM dNTPs (USB Corporation, Cleveland, OH), forward labeled and reverse primers (Table 1), 1 U AmpliTaq Gold DNA Polymerase (Applied Biosystems), and 0.16 mg/mL BSA (Sigma-Aldrich). PCR conditions for the multiplex assay are as follows: denaturation for 11 min at 95°C, amplification for 30 cycles of 45 sec at 94°C, 2 min at 59°C, and 1 min at 72°C, extension for 60 min at 60°C, and a final soak at 25°C.

### PCR product analysis

Samples were prepared by adding 1 μL of amplified product and 0.3 μL of GeneScan™ 500 LIZ internal size standard (Applied Biosystems) to 8.7 μL of Hi-Di™ (Applied Biosystems) for separation on the 16-capillary ABI 3130*xl *Genetic Analyzer (Applied Biosystems). A five dye matrix was established under the G5 filter with dyes 6FAM, VIC, NED, PET, and LIZ. POP-4™ (Applied Biosystems) was utilized on a 36 cm capillary array (Applied Biosystems) with 1× ACE buffer (Amresco, Solon, OH). Samples were injected electrokinetically for 10 sec at 3 kV. The STR alleles were separated at 15 kV at a run temperature of 60°C. Data from the 3130*xl *was analyzed using the GeneMapper *ID-X *v1.1 Software (Applied Biosystems). Bins and panels were generated in GeneMapper *ID-X *based on fragment length data from the sixty vervet monkey profiles using fixed bin allele sizes to determine allele calls. Calibration of repeat number to allele fragment length was determined by DNA sequencing.

### DNA sequencing

DNA samples were sequenced to determine the fragment length and number of correlating tetranucleotide repeat units. Sequencing primers were initially designed outside of the multiplex primer region; however, these primers did not always amplify the targeted region efficiently. This may be due to single-nucleotide polymorphisms (SNPs) located in the primer binding sequence or non-specific binding of the primer to other regions of the genome. As a result, the majority of primers used in the multiplex for genotyping were also used for sequencing due to difficulty in finding effective sequencing primers. At least two homozygote samples were sequenced for each STR locus to determine the corresponding number of repeats for each allele. The targeted repeat regions were amplified using 0.15 μM unlabeled forward and reverse primers (Table 2) using the PCR reaction specified in the PCR Amplification section with the following thermal cycling program: denaturation for 10 min at 95°C, amplification for 35 cycles of 1 min at 94°C, 1 min at 55-60°C (annealing temperature specific to individual primers), and 1 min at 72°C, extension for 45 min at 60°C, and a final soak at 25°C. Samples were treated with 2 μl of ExoSap-IT™ (USB Corporation) per 5 μl of PCR product to remove unincorporated primers and dNTPs and run on a thermocycler with the following conditions: 90 min at 37°C, 20 min at 80°C to inactivate the enzymes, and a final soak at 25°C. Samples were then sent to Eurofins MWG Operon for sequencing using BigDye^® ^Terminator v3.1 (Applied Biosystems).

**Table 2:** Sequencing primers used for sequence verification and repeat number determination

STR Marker	Sequencing Primers (5'-3')	Amplicon Size (bp)	Tm (°C)
D8S1106	F- GTTTACCCCTGCATCACTGG R- GGTGGCCTAACCAGAGTTGA*	267-291	55
DYS389	F - CCAACTCTCATCTGTATTATCTATG R - GTCTTATCTCCACCCACCAGA	321-337	60
D1S518	F - GCAGATCTTGGGACTTCTCG R - GTGTGGGCAACTGCATTAGAG	182-198	55
D6S1017	F - CTGGCACAGGATAGGTGCTC R - GATTGAACCAGATGGGAACGA	354-374	59
D17S1304	F - ACCATGTCCTCTGGTTCTGG R - GTTTCTTACAGGTGGGACTTGGTGAAA	197-213	60
D4S2408	F - TCATTTCCATAGGGTAAGTGAAAA R - GCCATGGGGATAAAATCAGA	336-360	60
D5S1467	F - GCCTAAGGTGGTGAATTGGA R - GAAGATGGCCCATTTCCATTT*	313-329	60
D19S245	F - GACCTGCAATCAGCCATTTT R - GTTCTTGCAGTCTGTGGCTTG	225-249	60

*New primers designed specifically for sequencing purposes. All other primers are original primers developed for the multiplex assay.

### Multiplex assay development

Optimization of the multiplex assay involved identifying an appropriate annealing temperature suitable for all primers included in the assay. Annealing temperature gradients ranging from 55°C to 60°C were used to screen the multiplex primers for optimal product formation. The concentration of vervet monkey genomic DNA (ranging from 1 ng to 10 ng) was also optimized based on the primer concentration and program cycle number. We found the most effective parameters for the multiplex to be the following: 59°C annealing temperature, 6 ng of vervet DNA, and 30 cycles for the amplification program. The primer concentrations were first optimized individually in monoplex reactions and then were combined into the final multiplex (Table 1). Primer concentrations were empirically determined based on peak height balance within the multiplex.

### Mixture analysis

Mixture samples containing genomic DNA extracted from Vero and CV-1 cells and Vero and HeLa cells were analyzed to determine the detection of low levels of contamination in Vero cells using the vervet multiplex assay. DNA from Vero and CV-1 cells were added to individual reactions with a final concentration of 6 ng of DNA in the following ratios 1:1, 2:1, 3:1, 5:1, 7:1, 9:1, and 10:1. Reciprocal reactions were also prepared using DNA from CV-1 and Vero cells. The same procedure was repeated using DNA from Vero and HeLa cells, followed by reciprocal reactions with DNA from HeLa and Vero cells.

### DNA analysis

The heterozygosity (H) values were calculated by dividing the number of heterozygotes at a locus into the total number of individuals. The probability of identity (P_I_) was calculated by the summation of the square of the genotype frequencies. The probability of a random match (PM) for a full profile was calculated by multiplying the inverse of each genotype frequency for each marker. In the formulas below, *n *is the number of samples, *P_i _*is the frequency of the *i^th ^*allele, *x_i _*is the frequency of the *i^th ^*genotype [19]. A NIST in-house Excel-based statistics program developed by David Duewer was used for these calculations.

$$Heterozygocity\left(H\right)=1-\overset{n}{\underset{i}{\sum }}P_{i}^{2}$$

$$Probabilityo\mathrm{fi}dentity\left(P_{I}\right)=\overset{n}{\underset{i=1}{\sum }}x_{i}^{2}$$

$$Probabilityofrandom\;match\left(PM\right)=\frac{1}{x_{i}}\left(forasinglemarker\right)$$

## Results and Discussion

### STR marker characterization

The vervet multiplex assay was developed by including eight human STR markers, all comprised of tetranucleotide repeats. The multiplex successfully identified sixty vervet monkey DNA samples received from the Washington National Primate Center resulting in unique profiles for each sample analyzed. Amplified PCR products for all eight loci were observed in every sample. Allele distributions were determined by screening the panel of vervet DNA samples and analyzing fragment lengths. The number of repeats was determined by sequencing at least two homozygote samples per locus and correlations were made between the fragment lengths and the number of repeats (Table 3).

**Table 3:** Correlating STR marker fragment length with repeat number

Human Marker	GenBank	Repeat Region	Allele Distribution (bp)	Number of Repeats
D8S1106	G09378	[ATAG]_n_	109, 121, 125, 129, 133	7, 10, 11, 12, 13
DYS389	G09600	[CTAT] _n_	321, 325, 329, 337	10, 11, 12, 14
D1S518	G07854	[AGAT] _n_	182, 186, 190, 194, 198	13, 14, 15, 16, 17
D6S1017	G08578	[ATCC] _n _[ACCC] _n _[ACCA] _n_	354, 358, 362, 366, 370, 374	10, 11, 12, 13, 14, 15
D17S1304	G07960	[TATC] _n_	197, 201, 205, 209, 213	10, 11, 12, 13, 14
D4S2408	G08341	[ATCT] _n _[ACCC][ATCT] _n _[ACCT] _n _[ATCT]	336, 344, 348, 352, 356, 360	13, 15, 16, 17, 18, 19
D5S1467	G08455	[AGAT] _n_	173, 181, 185, 189	8, 10, 11, 12
D19S245	L13119	[ATCT] _n _[ATCA][TCTA] _n_	225, 229, 233, 237, 241, 245, 249	16, 17, 18, 19, 20, 21, 22

The eight human STR markers incorporated in the multiplex assay with corresponding repeat region, fragment length (based on LIZ GeneScan™500 size standard), and number of repeats based on the DNA analysis from sixty-two vervet monkey samples. The correlation of the allele size and number of repeats was determined based on sequencing data.

**Figure 1:**
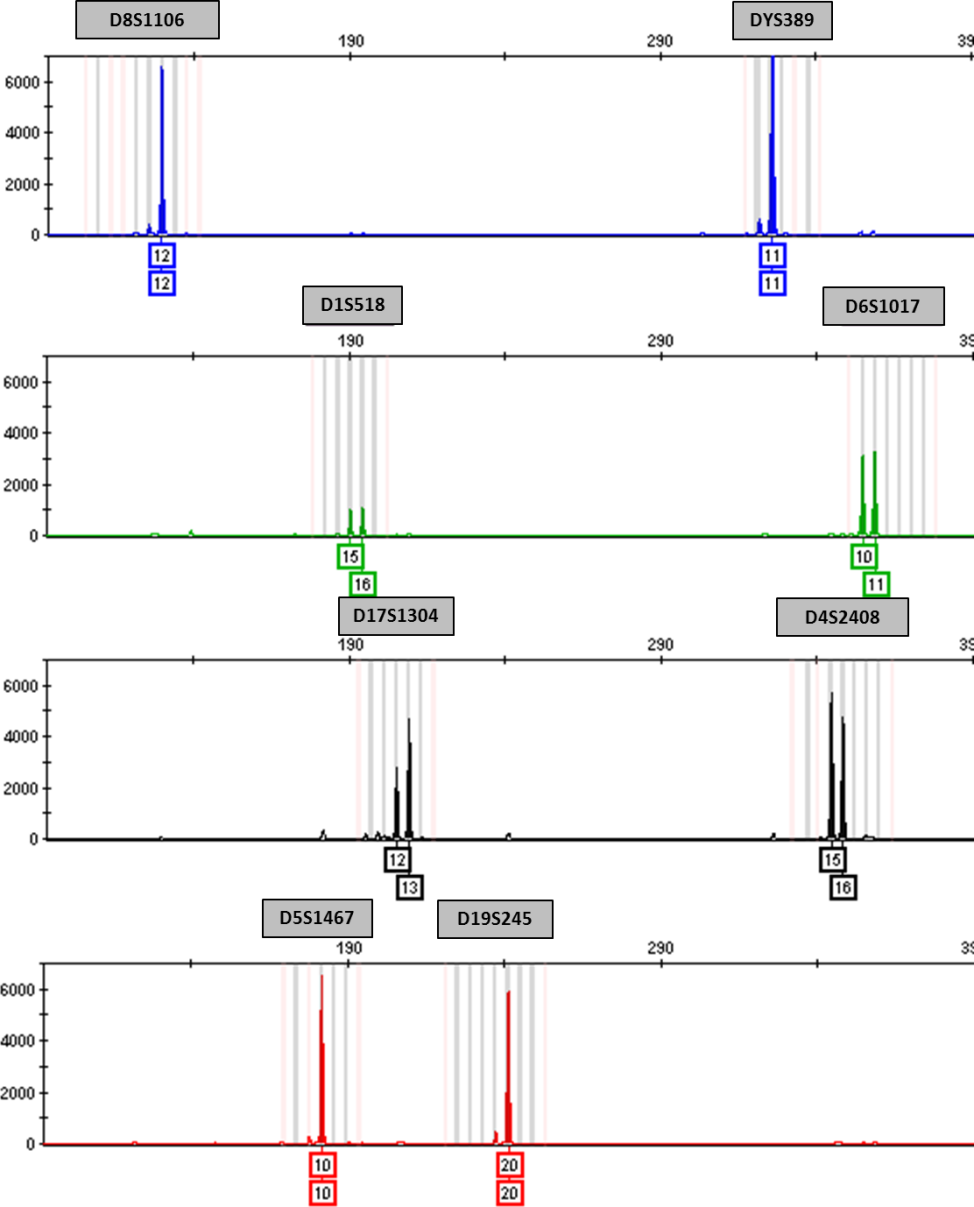
**Genetic profile of the Vero cell line using the vervet multiplex assay and bins and panels developed in GeneMapper *ID-X***. The blue, green, black, and red peaks in the electropherogram correspond to the fluorescently labeled forward primers for that STR marker (FAM, VIC, NED, and PET, respectively). Relative fluorescent units (RFUs) are depicted on the y-axis and fragment length on the x-axis. The number(s) below each peak represent the number of repeats at that locus. Vero peaks appear in the shaded bins and panels designated for each STR marker. The bins represent individual alleles for a specific marker.

**Figure 2:**
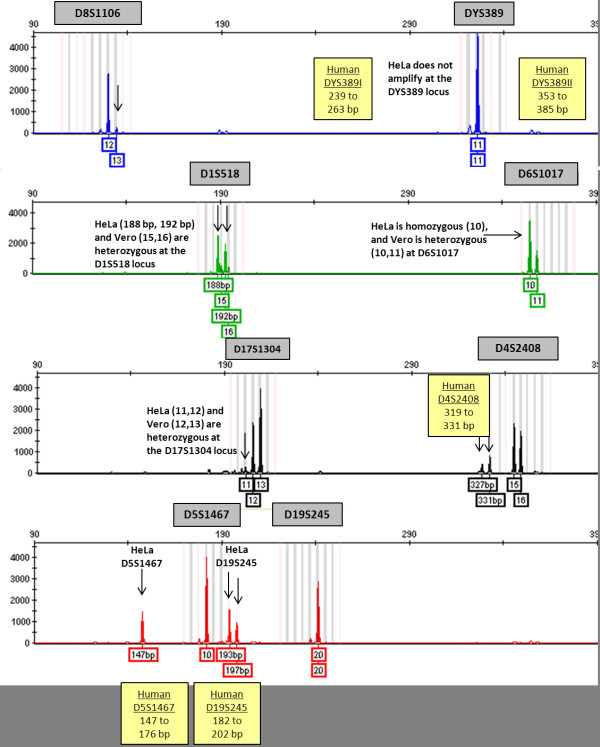
**Electropherogram representing a mixture of Vero and HeLa cell DNA (10:1 ratio, respectively)**. Genetic profiles were analysed using bins and panels developed in GeneMapper *ID-X*. Relative fluorescent units (RFUs) are depicted on the y-axis and fragment length on the x-axis in the electropherogram. Arrows depict HeLa alleles which may or may not appear in the bins and panels designated for Vero samples. Vero alleles appear in the shaded bins and panels designated for each STR marker. The number(s) below each peak represent the number of repeats at that locus. At the D4S2408, D5S4167, and D19S245 markers, HeLa DNA amplifies outside the observed vervet allele range and those values are represented as fragment lengths (bp) instead of repeat numbers. HeLa cells do not amplify at the DYS389 locus because they are of female origin. Human allele ranges that differ from vervet monkeys are illustrated in yellow boxes.

Four vervet cell lines, Vero, Vero76, CV-1 and COS-7, were also tested using the multiplex assay (Figure 1). Vero and Vero76 genomic DNA samples resulted in identical profiles, confirming that these two cell lines were derived from the same vervet individual [38]. Similarly, DNA extracted from COS-7 cells produced the same profile as the CV-1 cell line from which it was derived [39] (Table 4). Genomic DNA from HeLa cells (female human origin), a common cell culture contaminant, was also analyzed with the multiplex assay which resulted in an "irregular profile". The HeLa alleles were outside of the determined allele range for vervet monkeys in three of the loci (D4S2408, D5S1467, and D19S245) and no amplified product was detected at the DYS389 locus. This was expected because DYS389 is a human marker for the Y chromosome, found only in males, and HeLa cells are female in origin. In addition to HeLa cell DNA, ten human DNA samples from NIST SRM 2391b (eight components), N4124, and Control DNA 007 were analyzed using the vervet multiplex assay and amplified products were present for all eight loci, excluding DYS389 if the sample was of female origin. In the eleven human samples tested, amplicon sizes for the D19S245 marker ranged from 182 to 202 base pairs, 147 to 176 base pairs for the D5S1467 marker, and 319 to 331 base pairs for the D4S2408 marker (illustrated in Figure 2). These allele ranges correlate with the results found in HeLa DNA.

**Table 4:** Genetic profiles for Vero, Vero76, COS-7, CV-1 cells and two examples of human profiles using the vervet multiplex assay

	D8S1106	DYS389	D1S518	D6S1017	D17S1304	D4S2408	D5S1467	D19S245
Vero/Vero 76	12, 12	11, 11	15, 16	10, 11	12, 13	15, 16	10, 10	20, 20
CV-1/COS-7	11, 13	11, 11	13, 16	11, 12	11, 11	19, 19	11, 11	21, 21
HeLa	13, 13	NA**	(188 bp, 192 bp)*	10, 10	11, 12	(327 bp, 331 bp)*	(147 bp)*	(193 bp, 197 bp)*
N4124	12, 13	(251 bp, 373 bp)*	(192 bp, 196 bp)*	13, 14	12, 12	(322 bp)*	(147 bp, 176 bp)*	(189 bp, 193 bp)*

*Apparent size based on LIZ GeneScan™500 size standard. These values do not fall within the vervet allele distribution range or in the designated bins (developed using GeneMapper ID-X Software) for the vervet monkey and therefore are listed as sizes as opposed to repeat numbers.

**No amplified product was detected at this locus because it is of female origin.

In vervet monkeys, the DYS389 marker is amplified whether the individual is male or female. This data agrees with a previous study indicating the primers used to amplify the DYS389 marker yield PCR products in both male and female Japanese macaques, suggesting the locus is either autosomal in nature or has sequence homology on the X and Y chromosomes [40]. In humans, the primers for DYS389 amplify two products in male samples, DYS389I and DYS389II, and it was found that these products do not exist in Japanese macaques. The data from this study suggests that the same is true for vervet monkeys at this locus. PCR products for the DYS389 marker from Japanese macaques range from 320 to 360 bases, similar to the allele ranges observed in this study for vervets, and in human males they range from 239 to 263 (DYS389I) and 353 to 385 (DYS389II) bases. The differences in the amplified product size for the DYS389 locus can be useful in distinguishing between cell lines of vervet and human (male) origin.

### Sequencing repeat regions

The D4S2408 and D6S1017 loci were based on the human STR markers from a 26plex assay developed to aid in human identification [22]. Since the D4S2408 and the D6S1017 loci are incorporated in both the human 26plex and the vervet multiplex assays, the samples tested were hypothesized to have the same number of repeats at these loci when the two assays were compared using the same DNA samples; however, this was not the case. When genomic DNA from HeLa cells, Vero cells, and human N4124 was tested with the human 26plex the resulting repeats for the D6S1017 locus were (8, 8), (8, 9), and (11, 12), respectively. When the same DNA was amplified with the vervet multiplex for the D6S1017 locus the resulting repeats were (10, 10), (10, 11), and (13, 14), respectively. The vervet multiplex results have two additional repeats when compared to the 26plex data and this is due to differences found in the nomenclature of the repeat region motif. In the 26plex, the repeat motif is defined as ATCC and the flanking sequence ACCA ACCA does not vary within human DNA sequence at this marker. In vervet DNA, the flanking sequence does vary and is therefore incorporated in the designated repeat motif [ATCC]_n_[ACCC]_n_[ACCA]_n_. Differences in nomenclature also explain why the human 26plex assay and the vervet multiplex assay give different results at the D4S2408 locus when comparing the same DNA sample. The D4S2408 locus in the 26plex assay has a simple ATCT repeat in human sequence; however, in vervet sequence we observed a more complex repeat of [ATCT]_n_[ACCC][ATCT]_n_[ACCT]_n_[ATCT]. The flanking regions outside the repeat region are identical in both human and vervet sequence. Due to differences in length of the D4S2408 repeat region found in human and vervet DNA, human samples result in smaller fragment lengths of the repeat amplicon when compared to the vervet allele range in the multiplex assay.

### Evaluation of STR markers

High heterozygosity values are important in selecting STR markers for human identification purposes [41]. In general, markers with high heterozygosity values give a greater chance of discriminating between any two individuals. Observed heterozygosity values greater than 70% were used in the selection process for STR markers for human identification applications [19]. Observed heterozygosity values were measured for each of the markers in the vervet multiplex based on sixty-two vervet genomic DNA samples (Table 5). The two loci that resulted in the highest observed heterozygosity values are D19S245 (0.790) and D1S518 (0.710). The STR markers with the lowest observed heterozygosity values (0.532) were DYS389 and D4S2408. The D1S518 locus gave comparable results to those published by Newman *et al*. [30]. These researchers used a smaller sample size of fifty-five monkeys, and found five alleles associated with this locus with an observed heterozygosity value of 0.596. The heterozygosity value for D1S518 calculated in this study is higher (0.710) than the value calculated in the Newman paper and is likely due to the number and type of samples analyzed in each study. The probability of identity (P_I_) was calculated for each marker (Table 5). For example, the D8S1106 marker has a probability of 1 in 9 that any two vervet samples would match at this marker. Since the eight markers are all located on separate chromosomes, we can multiply the probability of identity for each marker to determine a random match. The probability of a random match using eight STR markers between any two vervet samples is 1 in 1.9 million.

**Table 5:** Observed heterozygosity and probability of identity values calculated for STR markers in the vervet multiplex samples

STR Marker	Number of Alleles	H (obs)	**P**_**I**_ (obs)
D8S1106	5	0.645	0.111
DYS389	4	0.532	0.226
D1S518	5	0.710	0.143
D6S1017	6	0.629	0.222
D17S1304	5	0.677	0.110
D4S2408	6	0.532	0.282
D5S1467	4	0.581	0.264
D19S245	7	0.790	0.082

Heterozygosity (H) and probability of identity (P_I_) values were calculated for each STR marker using data from sixty-two vervet monkeys.

### Species specificity of the assay and profile stability

To determine the specificity of the selected STR markers, genomic DNA from pig, mouse, hamster, rat, gerbil, baboon, rhesus, and cynomolgus monkey were tested using the vervet multiplex assay. DNA samples with concentrations ranging from 2 ng to 100 ng were added to individual reactions and samples were amplified according to the methods in the PCR amplification section. All eight non-vervet DNA samples resulted in incomplete or irregular profiles in the limited number of samples tested based on the bins and panels developed for the vervet monkey in GeneMapper *ID-X *(data not shown). To further explore the genetic diversity between these species would require screening of additional samples; however, this is beyond the scope of the study.

A multiplex assay that can detect contamination of other cell lines within a mixed sample would be useful. Contamination of Vero cells with another vervet cell line (CV-1) or a human cell line (HeLa) was analyzed using simulated mixture samples. Mixture ratios ranging from 1:1 to 10:1 consistently showed mixed profiles using the multiplex assay. Even at the highest dilution in Vero DNA (5.45 ng), low concentrations of HeLa and CV-1 DNA (0.55 ng) were detected within a Vero profile (See Figure 2). The inverse is also true. Samples containing low levels of Vero DNA (0.55 ng) produced a full Vero profile even with high levels of CV-1 DNA present and resulted in a partial profile with high levels of HeLa DNA. The partial profile consisted of six STR markers, D8S1106, DYS389, D1S518, D17S1304, D5S1467, and D19S245, and the remaining two loci, D4S2408 and D6S1017, resulted in peaks that were below the analytical threshold of 50 relative fluorescent units (RFU).

Genetic stability of the eight STR markers was evaluated over high cell passage number to ensure a stable profile will be generated as the cell line ages. DNA extracted from Vero cells harvested at passage numbers 7, 14, 22, 35, 43, 58, 63, and 69 were analyzed using the multiplex assay. After samples were amplified and separated based on fragment length, it was determined that there was complete concordance in the Vero profile up to 69 passages. These data suggest that the STR loci are stable with low mutation rates in the Vero cell line.

## Conclusions

A multiplex assay based on eight human STR markers with observed heterozygosity values ranging from 0.532 to 0.790 was developed, and the probability of a random match using these markers between any two vervet samples is approximately 1 in 1.9 million. There are several advantages of using an STR assay to genotype vervet cells and their derivatives including the ability to obtain more information from a multiplex, the rapid detection using PCR and capillary electrophoresis separation, and the relative low cost of materials and reagents.

The markers described in this assay can be useful to authenticate cell lines derived from individual vervet monkeys; however, vervet cells derived from the same individual will result in identical profiles. Additional specific assays are required to distinguish between cell lines derived from the same origin. For example, COS-7 and CV-1 cells result in the same genetic STR profile, so a test for SV40 T antigen could be used to differentiate COS-7 cells from CV-1 cells [39].

Genetic stability of the eight loci in this assay was shown to be consistent at high passage in the Vero cell line over a period of six months. This supports the findings of Chiong *et al*. where they concluded STR profiling of human bladder cancer cell lines does not change with passage number [42]. Parson *et al*. described genetic stability in some human leukemia cell lines and instability in others after passaging cells for one year and analyzing the DNA fingerprints using STR markers [43]. High cell passage number has been shown to increase the risk of alterations in DNA fingerprinting and best practices suggest cell identification at low passage numbers [44].

In addition to authenticating vervet cell lines, this assay may be a useful indicator for human cell line contamination since the assay is based on human STR markers. Human DNA amplifies at each marker; however, the fragment sizes are outside the observed vervet allele range at the following loci: D4S2408, D5S1467, D19S245, and DYS389. Human cell line contamination can be confirmed using validated STR markers [17]. The vervet multiplex assay was unable to generate complete profiles in the observed vervet allele range for more distantly related species, and we are currently developing additional markers to target several of these in an all-inclusive multiplex assay.

## Authors' contributions

JA carried out the cell work, PCR optimization, capillary electrophoresis, GeneMapper *ID-X *software analysis, and sample preparation for and analysis of the sequencing results. KC participated in the design and coordination of the study. CH participated in the multiplex optimization parameters. JA drafted the manuscript and CH and KC revised it critically for important intellectual content. All authors read and approved the final manuscript.
